# LncRNA DLEU1 is overexpressed in premature ovarian failure and sponges miR-146b-5p to increase granulosa cell apoptosis

**DOI:** 10.1186/s13048-021-00905-x

**Published:** 2021-11-05

**Authors:** Caihong Zheng, Shiwei Liu, Zhihong Qin, Xiaoqian Zhang, Yubao Song

**Affiliations:** 1 Department of Endocrinology, Shanxi Bethune Hospital, Taiyuan City, Shanxi Province 030032 People’s Republic of China; 2 Department of Endocrinology, Huhhot First Hospital, Huhhot, The Inner Mongolia, Autonomous Region 010020 People’s Republic of China; 3 Department of Endocrinology, Jincheng General Hospital, Jincheng City, Shanxi Province 048006 People’s Republic of China; 4 Second Department of General Surgery, Shanxi Provincial Cancer Hospital, No. 3, Xincun Worker’s Village, Xinghualing District, Taiyuan City, Shanxi Province 030013 People’s Republic of China

**Keywords:** DLEU1, POF, miR-146b-5p, Premature ovarian failure

## Abstract

**Background:**

miR-146b-5p has been reported to participate in premature ovarian failure (POF) in mice. However, its role in POF patients is unclear. We predicted that miR-146b-5p might interact with lncRNA DLEU1, a crucial player in ovarian cancer. We then explored the interaction between DLEU1 and miR-146b-5p.

**Methods:**

Expression of DLEU1 and miR-146b-5p in POF and control ovary tissues was determined by RT-qPCR. The subcellular location of DLEU1 in human KGN cells was analyzed using subcellular fractionation assays. The direct interaction between DLEU1 and miR-146b-5p was analyzed using RNA pull-down assays. The role of DLEU1 in miR-146a expression was analyzed using overexpression assay. Cell proliferation was analyzed using cell apoptosis assay.

**Results:**

Increased DLEU1 expression and decreased miR-146b-5p expression were observed in POF. DLEU1 directly interacted with MiR-146b-5p and was expressed in both nuclear and cytoplasm samples of KGN cells. In KGN cells, DLEU1 and miR-146b-5p failed to regulate the expression of each other. However, DLEU1 promoted cell apoptosis and reduced the inhibitory effects of miR-146b-5p on cell apoptosis.

**Conclusions:**

DLEU1 is overexpressed in POF and sponges miR-146b-5p to increase KGN cell apoptosis.

## Introduction

Premature ovarian failure (POF), also known as primary ovarian insufficiency, is a common cause of infertility and is characterized by hypoestrogenism, elevated gonadotropins, and amenorrhea [1, 2]. POF is a rare clinical condition that only affects about 1–5 out of 1000 women younger than 40 years [3]. Early POF patients usually present with abnormally high FSH level (> 10 IU/L), regular menstruation, but reduced fertility. With the development of POF, menopause will occur, leading to ovarian primordial follicle pool premature depletion [4, 5]. At present, POF is still a non-reversible pathological change [6, 7]. At present, only 5% of POF patients can conceive spontaneously and experience a normal pregnancy [8]. Therefore, novel approaches are needed.

Understanding the molecular mechanism of POF may provide novel insights into the development of novel anti-POF strategies [9–11]. With the increased elucidation of genes involved in this disease, some molecular mediators, such as Ntrk2/Kiss1r pathway, show promising potentials in the treatment of POF [12]. However, POF-targeted therapy is still under research. More targets, especially those with high safety and efficiency, are still needed [9–12]. Besides protein players, progression of POF also requires the involvement of non-coding RNAs, such as miRNAs and lncRNAs [13], suggesting that ncRNAs are a gold mine for the development of novel targets to treat POF. MiR-146b-5p has been reported to participate in POF in mice [14]. However, its role in POF patients is unclear. We predicted that miR-146b-5p may interact with lncRNA DLEU1, a crucial player in ovarian cancer [15], and explored the interaction between DLEU1 and miR-146b-5p.

## Materials and methods

### Research patients

Study population included both POF patients (*n* = 49) and controls (*n* = 49). Granulosa cell (GC) tissues were donated by both groups of participants at Shanxi Provincial Cancer Hospital after the Ethics Committee of the present study approved this study. At this hospital, intracytoplasmic sperm injection, embryo transfer, and in vitro fertilization were performed on POF patients. Primary granulosa cells were isolated from the follicular fluid of POF patients on the day of oocyte collection. Controls had normal serum FSH levels (< 10 IU/L) and menstrual cycles and received infertility treatment due to male factors of tubal obstruction. The diagnosis of POF was based on the following criteria: 1) > 10 IU/L of basal serum FSH, 2) younger than 40 years, and 3) normal menstrual cycles (23–35 days). Baseline data of both patients and controls were presented in Table 1. All POF patients and controls signed informed consent.

**Table 1:** Baseline data of both patients and controls

Variables	Control (n = 49)	POF (***n*** = 49)
Age (y)	29.98 ± 3.18	30.34 ± 4.61
basal FSH (IU/L)	5.98 (4.89, 7.11)	14.02 (11.99, 20.23)**
BMI (kg/m2)	22.03 (19.72, 22.82)	22.10 (19.21, 26.37)
basal LH (IU/L)	5.52 (3.81, 7.78)	5.47 (4.01, 8.62)
basal E2 (pg/mL)	29.82 (23.08, 43.45)	30.98 (12.56, 43.12)
AMH (ng/mL)	3.34 (2.23, 5.11)	0.42 (0.28, 0.89)**

**,*p* < 0.01

### KGN cells, primary granulosa cells, and transfections

KGN cells (human granulosa-like tumor cells) were from RIKEN BioResource Center (Tsukuba, Japan) and cultured in DMEM/F-12 media (HyClone) containing 10% FBS at 37 °C in a humidified incubator with 5% CO_2_. Primary granulosa cells were isolated from follicular fluid of POF patients through centrifugation and cultured at the same conditions as KGN cells.

DLEU1 and miR-146b-5p were transiently overexpressed in KGN cells by transfecting DLEU1 vector or MiR-146b-5p mimic using Lipofectamine 2000 (Invitrogen). Each transfection was performed with 10^7^ cells and 12 μg vector or 40 nM mimic. Cell culture of un-transfected cells was performed until the end of transfections to serve as a control. NC miRNA or empty vector transfection was also included as negative control (NC). The subsequent experiments were done 48 h later.

### RNA preparations of RT-qPCR

GC tissue samples and KGN cells were used to isolate total RNAs with PicoPure™ RNA Isolation Kit (Thermo Fisher Scientific). RNA samples were digested with DNase I (Invitrogen) to remove DNA contamination. RNA integrity and concentrations were determined using Bioanalyzer. Only RNA samples with a RIN value higher than 8 were considered for subsequent RT-qPCRs.

With 5000 ng total RNA as template, cDNA samples were prepared through reverse transcriptions and subjected to qPCRs to determine the expression of DLEU1 and miR-146b-5p. Internal controls for DLEU1 and miR-146b-5p were 18S rRNA and U6, respectively. Ct values of target genes were normalized to corresponding internal controls using the 2^-ΔΔCt^ methods.

### Cell fractionation assay

PARIS kit (Invitrogen) was used to prepare nuclear and cytoplasm samples from KGN cells by centrifugation at 1200 g for 10 min. Other operations, including further nuclear lysis, were performed according to the manufacturer’s instructions. The two fractions were then used in RNA isolation and RT-qPCRs to determine DLEU1 expression.

### RNA pull-down assay

In vitro transcripts of full-length DLEU1 and NC RNAs were prepared with MEGAscript T7 transcription kit (Invitrogen) and labeled with biotin at 3′ ends using Pierce™ RNA 3′ End Biotinylation Kit (Thermo Fisher Scientific). The two labeled RNAs (Bio-NC and Bio- DLEU1) were transfected into KGN cells. At 48 h post-transfection, cells were lysed on ice for 30 min. RNA samples were pulled down using magnetic beads from these two samples, reverse transcribed into cDNAs, and subjected to PCRs to determine DLEU1 expression.

### Cell apoptosis analysis

KGN cells were harvested 48 h post-transfection and cultured in serum-free fresh media for 48 h. After that, cells were washed with PBS, resuspended in binding buffer, stained with FITC labeled Annexin-V and PI, and subjected to FACS Caliber flow cytometry to analyze cell apoptosis.

### Statistical analysis

Two participant groups were compared by unpaired t test. ANOVA Tukey’s test was used to compare multiple independent groups. A *p* < 0.05 was statistically significant.

## Results

### Exploration of DLEU1 and miR-146b-5p expression in POF

**Fig. 1:**
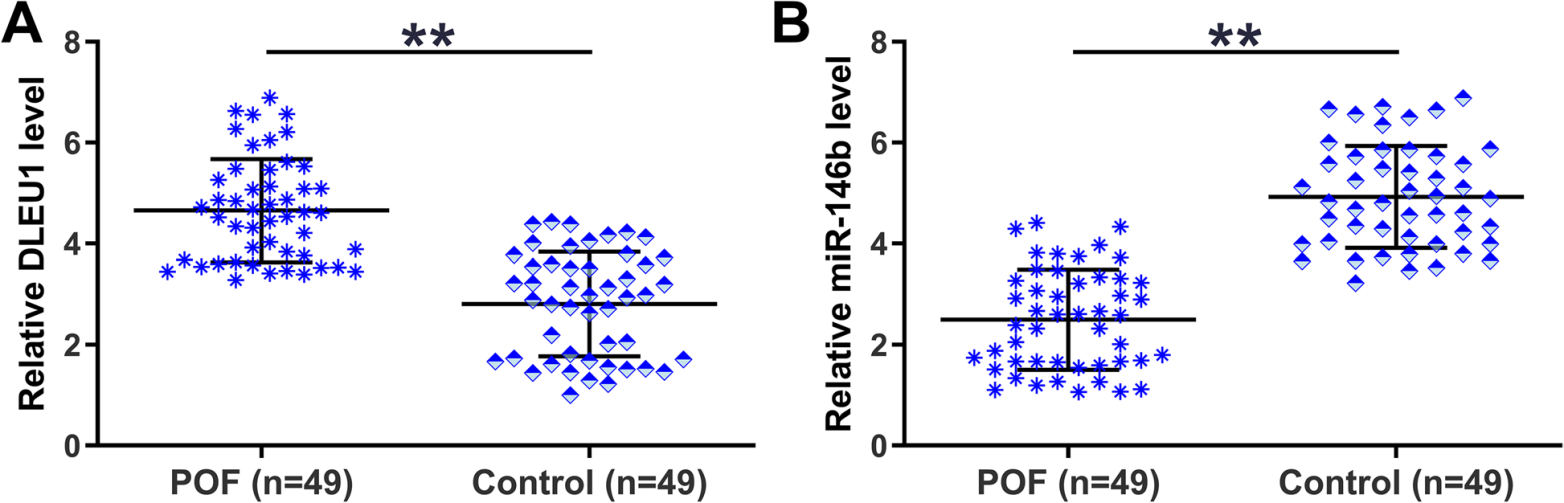
Exploration of DLEU1 and miR-146b-5p expression in POF. GC samples from both POF patients (*n* = 49) and controls (*n* = 49) were used for total RNA isolation. Total RNA samples were subjected to RTs and qPCRs to explore the differential expression of DLEU1 (**A**) and miR-146b-5p (**B**) in POF. **, *p* < 0.01

Total RNAs were isolated from GC samples from both POF patients (*n* = 49) and controls (*n* = 49) and subjected to RTs and qPCRs to explore the differential expression of DLEU1 and miR-146b-5p in POF. The results showed that DLEU1 expression was increased (Fig. 1A, *p* < 0.01), and miR-146b-5p expression was decreased (Fig. 1B, *p* < 0.01) in POF. Therefore, DLEU1 and miR-146b-5p might be involved in POF.

### Direct interaction between DLEU1 and miR-146b-5p and subcellular location of DLEU1 in KGN cells

**Fig. 2:**
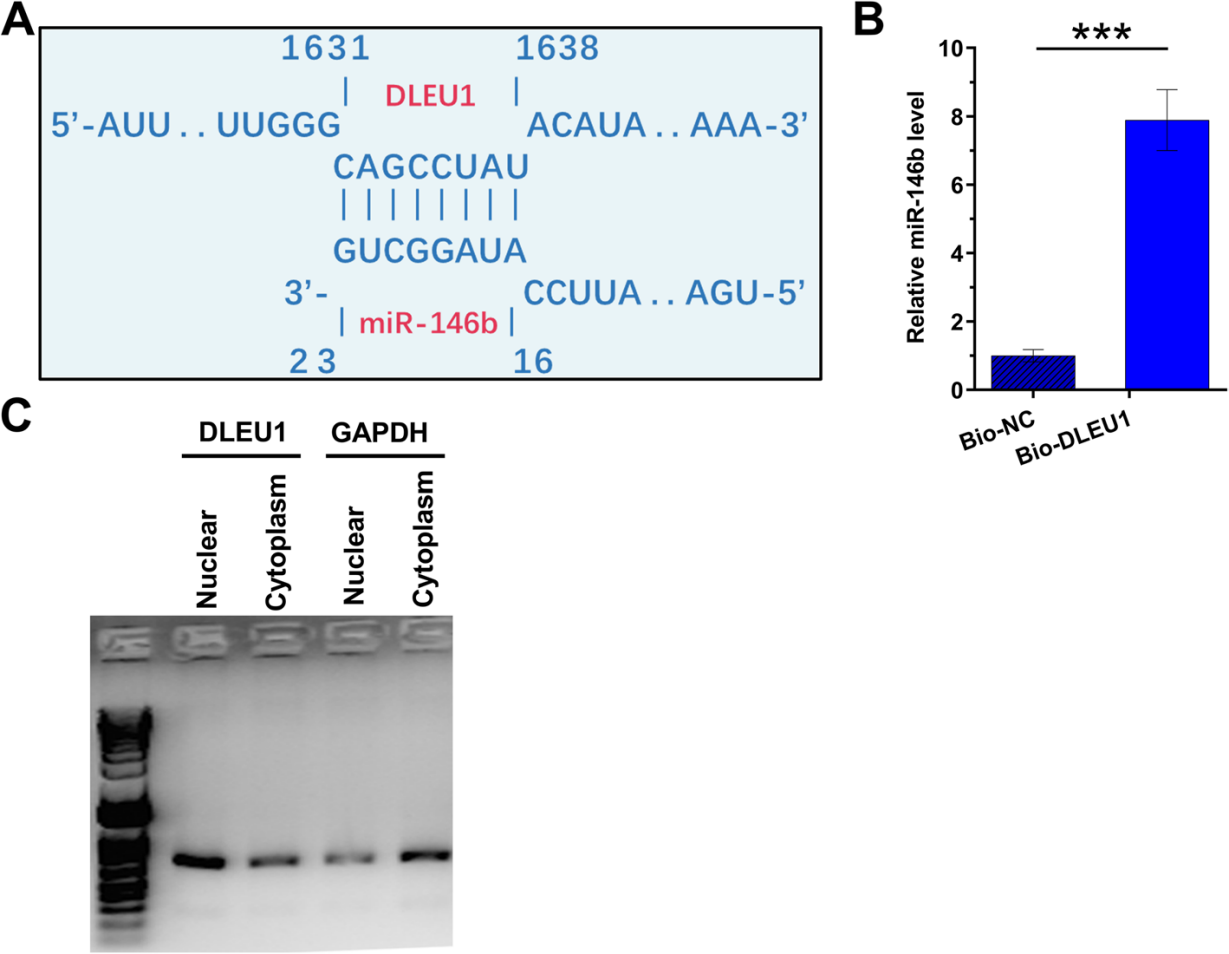
Exploration of the direct interaction between DLEU1 and miR-146b-5p and subcellular location of DLEU1 in KGN cells. IntaRNA 2.0 (**A**) was used to predict and RNA pull-down assay (**B**) was performed to validate the direct interaction between DLEU1 and miR-146b-5p. The cellular fractionation assay was carried out to analyze the subcellular location of DLEU1 in KGN cells (**C**). ***, *p* < 0.001

IntaRNA 2.0 and RNA pull-down assay were performed to predict and validate the direct interaction between DLEU1 and miR-146b-5p. Our prediction showed that DLEU1 and miR-146 could form base pairs (Fig. 2A). RNA pull-down assay showed that, compared to Bio-NC pull-down group, Bio-DLEU1 pull-down group exhibited a significantly increased miR-146b-5p expression, which validated the direct interaction between them (Fig. 2B, *p* < 0.001). To further confirm the direct interaction between DLEU1 and mature miR-146b-5p, which is only localized to the cytoplasm, the subcellular location of DLEU1 in KGN cells was analyzed using cellular fractionation assay. DLEU1 could be detected in both nuclear and cytoplasm fractions of KGN cells (Fig. 2C).

### Crosstalk between DLEU1 and miR-146b-5p

**Fig. 3:**
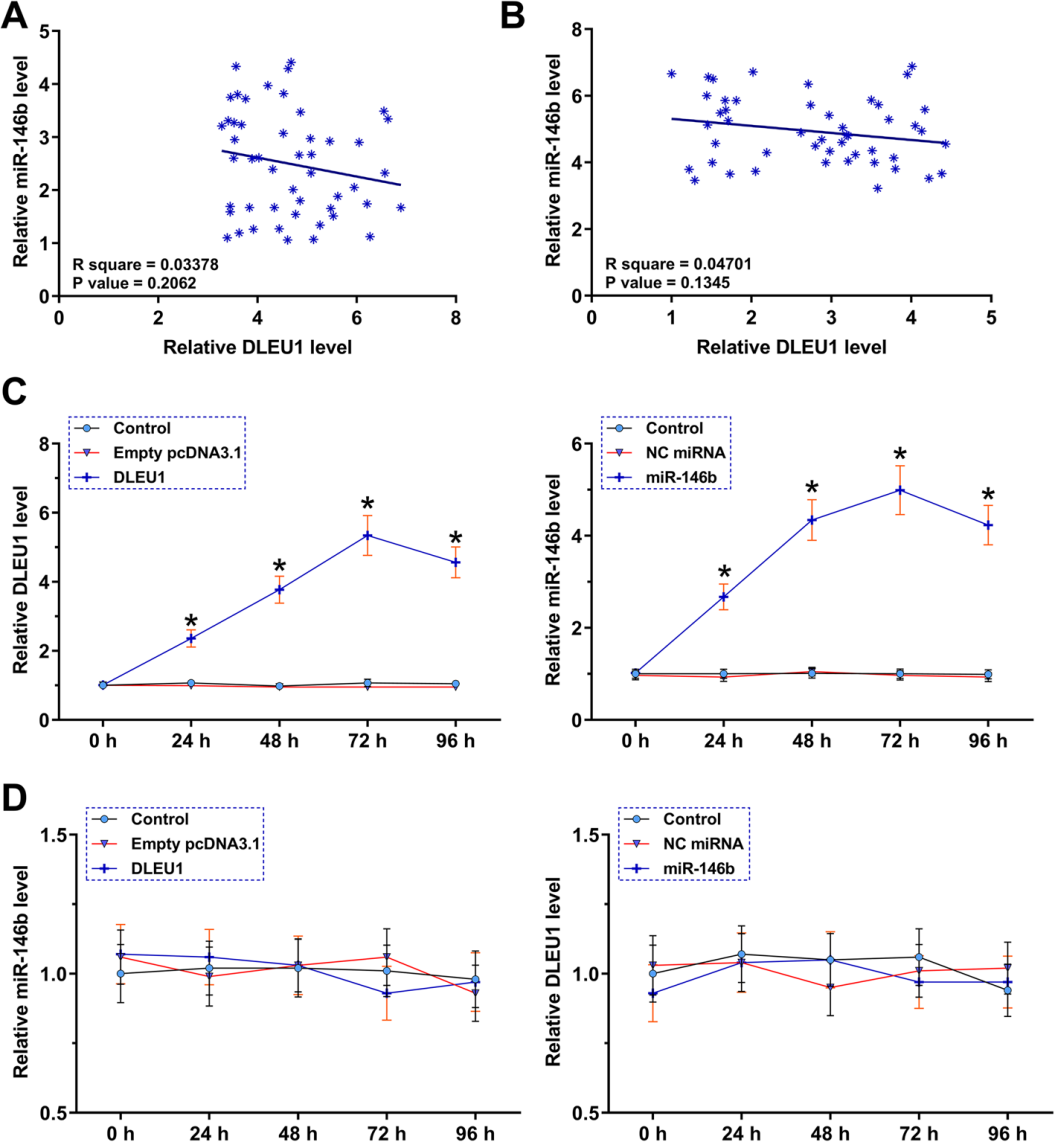
Exploration of the crosstalk between DLEU1 and miR-146b-5p. Correlations between DLEU1 and miR-146b-5p across both POF (**A**) and control (**B**) GC samples were analyzed by Pearson’s correlation coefficient. DLEU1 and miR-146b-5p were overexpressed in KGN cells, and the transfections were confirmed by RT-qPCR every 24 h until 96 h (C). The roles of DLEU1 and miR-146b-5p in the expression of each other in KGN cells were analyzed with RT-qPCRs (D). *, *p* < 0.05

Correlations between DLEU1 and miR-146b-5p across both POF (Fig. 3A) and control (Fig. 3B) GC samples were analyzed by Pearson’s correlation coefficient. Interestingly, DLEU1 and miR-146b-5p were not significantly correlated across both samples. To further analyze their interactions, DLEU1 and miR-146b-5p were overexpressed in KGN cells. Their overexpression was confirmed by RT-qPCR every 24 h until 96 h (Fig. 3C, *p* < 0.05). In KGN cells, DLEU1 and miR-146b-5p failed to regulate the expression of each other (Fig. 3D).

### Roles of DLEU1 and miR-146b-5p in the apoptosis of KGN cells and primary granulosa cells

**Fig. 4:**
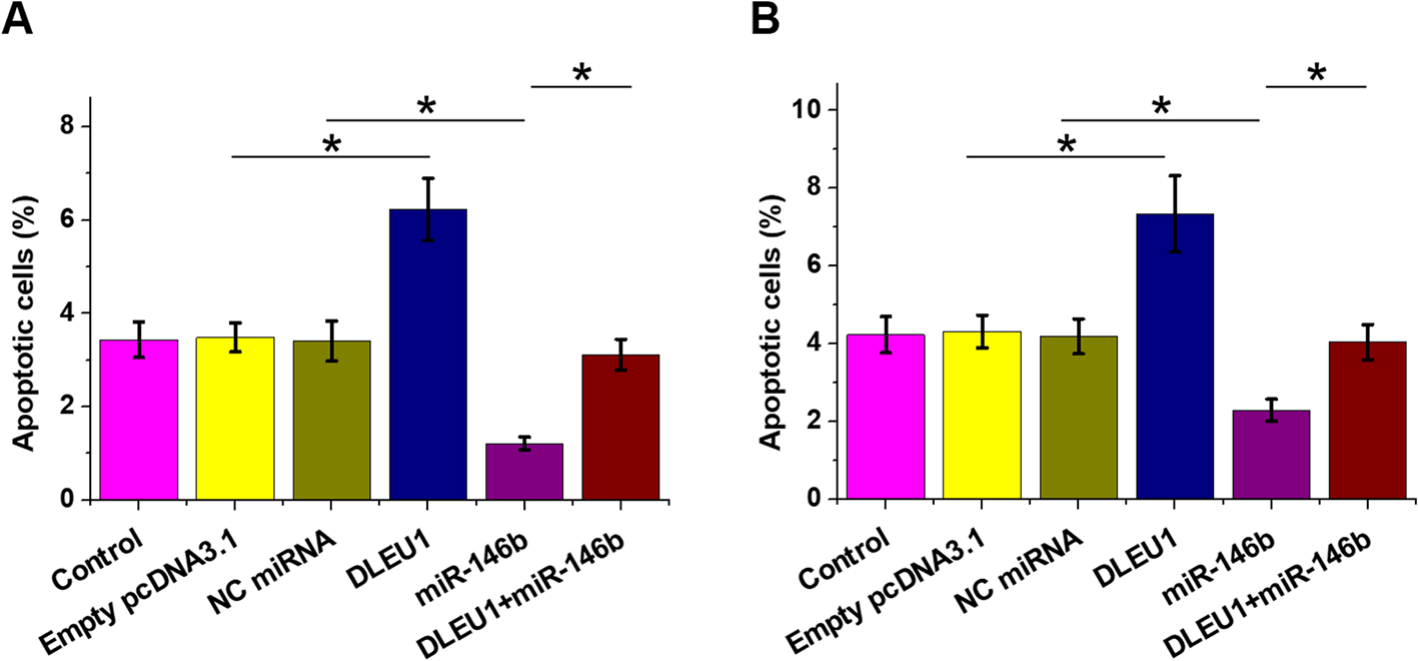
Analysis of the role of DLEU1 and miR-146b-5p in the apoptosis of KGN cells and primary granulosa cells. Cell apoptosis was carried out to study the role of DLEU1 and miR-146b-5p in the apoptosis of KGN cells (**A**) and primary granulosa cells (**B**). *, *p* < 0.05

Cell apoptosis was carried out to study the role of DLEU1 and miR-146b-5p in the apoptosis of KGN cells (Fig. 4A) and primary granulosa cells (Fig. 4B). DLEU1 promoted cell apoptosis, while MiR-146b-5p decreased cell apoptosis. In addition, DLEU1 reduced the inhibitory effects of miR-146b-5p on cell apoptosis (*p* < 0.05).

## Discussion

POF is a common clinical disorder with complex molecular mechanisms. In this study, we explored the expression of DLEU1 and miR-146b-5p in POF and analyzed their roles in regulating granulosa cell apoptosis. Our study suggested that DLEU1 and miR-146b-5p may serve as a potential target for POF.

The role of DLEU1 has only been explored in ovarian cancer [15]. It was observed that DLEU1 is under-expressed in ovarian cancer and regulates TFAP2A expression via miR-429, thereby suppressing tumorigenesis [15]. The involvement of DLEU1 in POF has not been reported previously. Granulosa cells (GCs) as follicular somatic cells promote folliculogenesis by secreting steroids and providing essential nutrients [16, 17]. In effect, GC dysfunction and increased GC apoptosis in POF contribute to disease progression. In this study, we showed that DLEU1 expression was increased in POF patients, and DLEU1 overexpression increased GC apoptosis. Therefore, DLEU1 overexpression in POF might promote disease progression by increasing cell apoptosis and DLEU1 silencing might serve as a potential target to treat POF.

MiR-146b-5p was reported to participate in POF in mice by suppressing γH2A phosphorylation and inactivating Dab2ip/Ask1/p38-Mapk signaling [14]. However, the role of miR-146b-5p in POF patients is unclear. This study showed the decreased miR-146b-5p expression in POF patients. In addition, miR-146b-5p overexpression decreased GC apoptosis. Therefore, miR-146b-5p plays a protective role in POF by suppressing cell apoptosis and upregulating miR-146b-5p expression might be applied in the clinical treatment of POF. The key finding of the present study is that DLEU1 could directly interact with miR-146b-5p, and DLEU1 could be detected in both nuclear and cytoplasm samples of GCs. Interestingly, DLEU1 and miR-146b-5p could not regulate the expression of each other, while DLEU1 suppressed the role of miR-146b-5p in cell apoptosis. The function of lncRNAs is to sponge miRNAs to suppress their function but may not affect their expression levels. Based on the data, we speculated that DLEU1 could sponge miR-146b-5p in the cytoplasm to promote GC apoptosis, thereby promoting POF.

## Conclusion

DLEU1 is overexpressed in POF and miR-146b-5p was downregulated in POF. DLEU1 may sponge miR-146b-5p in the cytoplasm to promote GC apoptosis, thereby promoting POF.

## Data Availability

The data are not publicly available due to their containing information that could compromise the privacy of research participants, but are available on request from the corresponding author.
